# Supplementary material for: Physical constraints and functional plasticity of cellulases
Source: Nat Commun. 2021 Jun 22;12:3847. doi: 10.1038/s41467-021-24075-y (PMC8219668; doi:10.1038/s41467-021-24075-y)
Supplement: Supplementary file 1 — Supplementary information [file 41467_2021_24075_MOESM1_ESM.pdf]

Supplementary Information

Physical constraints and functional plasticity of cellulases

Jeppe Kari[1#], Gustavo A. Molina[1#], Kay S. Schaller[1], Corinna Schiano-di-Cola[1], Stefan J. Christensen[1], Silke F. Badino,[1], Trine H. Sørensen[3], Nanna S. Røjel[2], Malene B. Keller[4], Nanna Rolsted Sørensen[2], Bartlomiej Kolaczkowski[2], Johan P. Olsen[3], Kristian B. R. M. Krogh[3], Kenneth Jensen[3], Ana M. Cavaleiro[3], Günther H. J. Peters[5], Nikolaj Spodsberg[3], Kim Borch[3] and Peter Westh[1]*

[1] Department of Biotechnology and Biomedicine, Technical University of Denmark, Søltofts Plads, 2800 Kongens Lyngby, Denmark, [2]Department of Science and Environment, Roskilde University, Universitetsvej 1, 4000, Roskilde, Denmark, [3]Novozymes A/S, Krogshøjvej 36, DK-2880, Bagsværd, Denmark, [4]Department of Geosciences and Natural Resource Management, University of Copenhagen, Rolighedsvej 23, 1958 Frederiksberg C, Denmark, [5]Department of Chemistry, Technical University of Denmark, Kemitorvet 207, 2800 Kongens Lyngby, Denmark.

*[#]Equal contributors, *Corresponding author: Email: petwe@dtu.dk ; Telephone: + 45 45 25 26 41*

Supplementary note 1 | Validity of the Michealis-Menten equation for cellulase kinetics

In this study we used the Michaelis-Menten (MM) equation to analyse the steady-state kinetics of 83 different cellulases. To test whether the simple MM-model was adequate for the current system, we derived a novel steady-state rate equation for a detailed model of the best studied cellulase (TrCel7A). We showed that the steady-state equation of the complex model can be expressed as a MM-equation and that the apparent MM-parameters for the complex model only gave rise to a scaling relationship when the rate-constant for the unbinding was changed (similar to what was suggested in the main text). The kinetic modelling shows that the simple MM-scheme may be regarded as a composite reaction scheme for the system studied here. This support the use of the MM-model for comparative analysis of a large set of cellulases of which there is little to no information about their molecular steps and underlying rate-constants.

The best studied cellulase is the processive cellobiohydrolase from the fungus *Trichoderma reesei* (TrCel7A) and much is known about its elementary steps in the overall hydrolysis of cellulose (1). We will use this enzyme as an example of how a detailed mechanism may be condensed into a Michaelis-Menten type of model and from this induce the generality of the MM-model for the other cellulases.

The molecular steps for TrCel7A have been reviewed in detail by Payne et al. (2) and include:

1. Adsorption of the enzyme to the cellulose surface.
2. Threading of an accessible free cellulose chain end into the active site tunnel.
3. Processive catalysis, which includes 3.1) hydrolysis of the glycosidic bond, 3.2) product expulsion from the product site and 3.3) sliding to form the next Michaelis-complex.
4. Dissociation from the surface.

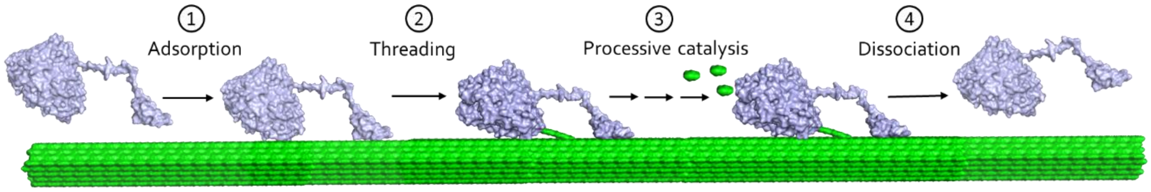

Supplementary figure 1*. Schematic illustration of the different molecular steps involved in the processive hydrolysis of cellulose by cellobiohydrolases.*

Step 1 and 2 in supplementary figure 1 have recently been investigated for TrCel7A (3,4). In these studies, time-resolved measurements of the threading and adsorption were used to elucidate the rate constants for the formation of a productive enzyme-substrate complex. In both cases, the threading/dethreading (step 2 in supplementary figure 1) was found to be slow compared to the fast dynamic of the adsorption/desorption step (step 1 in supplementary figure 1). This implies that the adsorbed but unthreaded enzyme rapidly inter-convert compared to other (slower) steps in the reaction mechanism and hence may be assumed to be in (rapid) equilibrium with respect to the threaded enzyme complex at all time. The next step (step 3 in supplementary figure 1) is what has been termed the *processive cycle* (5). This step is actually not one but several elementary steps, which a processive cellulase cycles through each time it moves productively (produces product) in the forward reaction path. The speed at which TrCel7A moves forward in its processive cycle has been measured to be 7 nm/s by high-speed atomic force microscopy (6). Since the product of TrCel7A is cellobiose, with a length of 1 nm, this translates into an overall rate constant for the processive cycle of 7 s^{-1}, which also matches values found from pre-steady-state measurements (7,8). In a recent study by Knott et al. (9) it was shown that the slowest step in the processive cycle of TrCel7A was the actual bond cleavage. By transition state path sampling, the authors found that the first step (glycosylation) in the two-step retaining catalytic mechanism, was the slowest step with a theoretical rate constant of 11 s^{-1}. This value is remarkably close to the 7 s^{-1} found by Igarashi et al. (6) and supports the hypothesis that the actual bond cleavage is the slowest step in the processive cycle (step 3 in figure S1). Since the processive cycle consists of a sequence of first-order elementary steps, the slowest step (glycosylation) will dominate at steady-state (10) and we will call this step *catalysis*. Due to the processive nature of the enzyme, the *catalysis "*step" is repated *n* times, where *n* is the processivity number of the enzyme. It has been shown that for TrCel7A, *n* is limited by the substrate (11) since the experimentally determined processivity is much smaller than the inherent processivity of the enzyme. This phenomenon may also be seen from pre-steady-state measurements, where the build-up of unproductive enzymes give rise to so-called burst kinetics (7,8,12-14). Real-time imaging of TrCel7A have shown that the build-up of unproductive enzyme is due to irregularities on the substrate that hinder further processive movement (6).

Based on the information above we can write a microkinetic scheme for the most important elementary steps in the overall hydrolysis of cellulose by TrCel7A (see supplementary scheme S1).

$$E + S_i \underset{k_{-1}}{\overset{k_1}{\rightleftarrows}} ES_{adsorb} \underset{k_{-2}}{\overset{k_2}{\rightleftarrows}} ES_{thread} \xrightarrow{n*k_3} nP + ES_{np} \xrightarrow{k_{-2}} E + S_{i-n} \quad \text{(supplementary Scheme 1)}$$

In supplementary table 1 we have summarized previously determined rate constants for all the steps in the supplementary scheme 1.

In the following, we will illustrate how the more realistic but complicated model would effect the apparent parameters and how scaling between apparent K_M and k_{cat} is restricted to the dissociation/dethreading event.

***Supplementary table 1**. Experimentally determined rate constants and processivity number for TrCel7A. The rate-constants refer to the reaction steps defined in supplementary scheme 1.*

Reaction steps	***Rate constant***	***reference***
Adsorption	$k_1 \sim 0.1\ s^{-1}\ \mu M^{-1}$	(3)
Desorption	$k_{-1} \sim 0.1\ s^{-1}$	(3)
Threading	$k_2 \sim 0.05\ s^{-1}$	(3,4)
Dissociation/Dethreading	$k_{-2} \sim 0.01\ s^{-1}$	(3,7)
Processive catalysis	$k_3 \sim 5\ s^{-1}$	(7,15)
	Parameter	
Processivity	n~10	(16)

We derived a steady-state rate equation for the reaction (supplementary scheme 1) using the two-Step computer-assisted method developed by Fromm and Fromm (17). In the derivation, we assumed rapid equilibrium with the free and adsorbed enzyme (step 1) which (as explained above) has been found to be a valid approximation. We also assumed that the substrate was in excess over the enzyme (S_0>>E_0) and that the total enzyme concentration was constant (mass conservation);

$$E_0 = E + ES_{adsorb} + ES_{thread} + ES_{np}$$

The differential equation was setup and solved in MATLAB using the two-Step computer-assisted method and the result was the steady-state rate equation given in supplementary eq. 1

$$v = \frac{E_0 S_0 \dfrac{n k_{-2} k_3 k_2}{(k_3 + k_{-2})(k_2 + k_{-2})}}{S_0 + \dfrac{k_{-1} k_{-2}(k_3 + k_{-2}) + k_3 k_2 k_{-2}}{k_1 (k_3 + k_{-2})(k_2 + k_{-2})}} \quad \text{(supplementary eq. 1)}$$

Supplementary equation 1 can be simplified to a Michealis-Menten type of equation

$$v = \frac{E_0 S_0\, {}^{app}k_{cat}}{S_0 + {}^{app}K_M} \quad \text{(Supplementary eq. 2)}$$

Where

$${}^{app}k_{cat} = \frac{n k_{-2} k_3 k_2}{(k_3 + k_{-2})(k_2 + k_{-2})} \quad \text{(Supplementary eq. 3)}$$

$${}^{app}K_M = \frac{k_{-1} k_{-2}(k_3 + k_{-2}) + k_3 k_2 k_{-2}}{k_1 (k_3 + k_{-2})(k_2 + k_{-2})} \quad \text{(Supplementary eq. 4)}$$

Since the dethreading step is known to be much slower than both the catalytic step and the threading step (see supplementary table 1), we may simplify the apparent MM-parameters as follows

$$^{app}k_{cat} \sim nk_{-2} \quad \text{for } k_{-2} << k_2 \wedge k_3 \qquad \text{(Supplementary eq. 5)}$$

$$^{app}K_M \sim \frac{k_{-2}(k_{-1}+k_2)}{k_2 k_1} \quad \text{for } k_{-2} << k_2 \wedge k_3 \text{ (Supplementary eq. 6)}$$

As seen from supplementary eq. 5 and 6, the only rate-constant that is present in both the apparent k_{cat} and K_M is the rate-constant for the dethreading. Hence, direct scaling between the two apparent MM-parameters would only be observed if the dethreading is changed. To test this prediction, we also simulated supplementary eq. 1 (with no additional assumption), where each parameter in the equation was changed to a value 10-times higher or 10-times lower than the initial value. The initial value was the most likely parameter extracted from literature (see supplementary table 1). A total of 10 values where scanned for each parameter and the step size between each value was logarithmic. The result of this analysis is shown in supplementary figure 2.

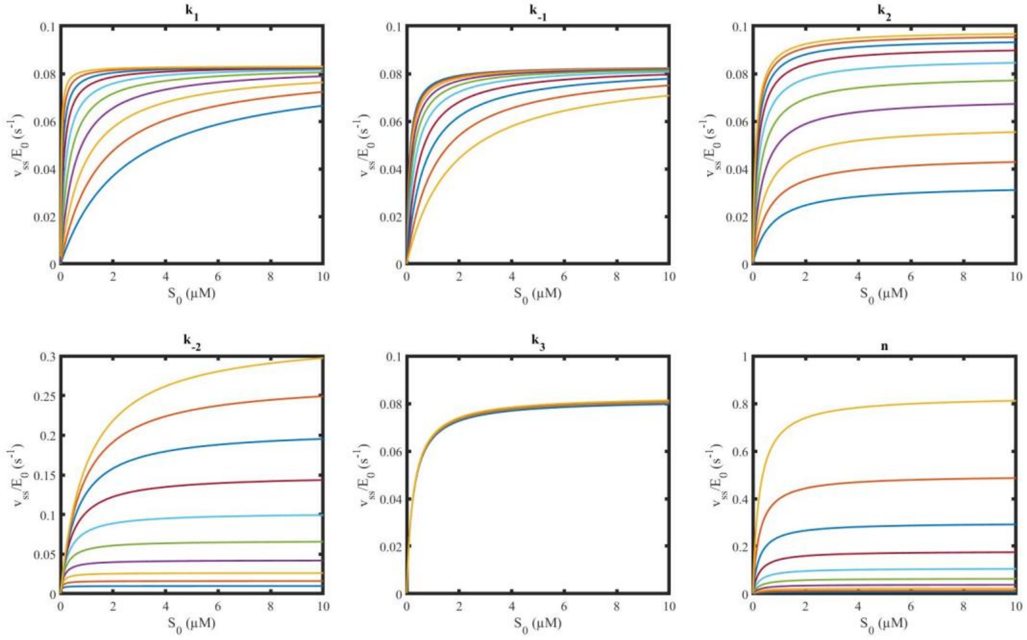

Supplementary figure 2*. Sensitivity analysis of supplementary eq. 1. Supplementary equation 1 was simulated as a function of substrate concentration (0→10 µM) using the parameters given in supplementary table 1 as initial input. The enzyme concentration was constant (0.1 µM) in all simulations. Each of the 6 subplot shows how the rate equation changes when all parameters are kept constant, except the one given by the title of the subplot. Each parameter was scanned individually with 10 different values (log-scaled step size) spanning from 10 times smaller to 10 times larger than the value reported in supplementary table 1.*

Since the steady-state solution given in supplementary eq. 1 can be expressed as a simple MM-equation (see supplementary eq. 2) we fitted the MM-equation to all the curves shown in supplementary figure 2 to obtain apparent MM-parameters for all the 6x10=60 saturation curves. The apparent k_{cat} (V_{max}/E_0) and K_M are shown in supplementary figure 3.

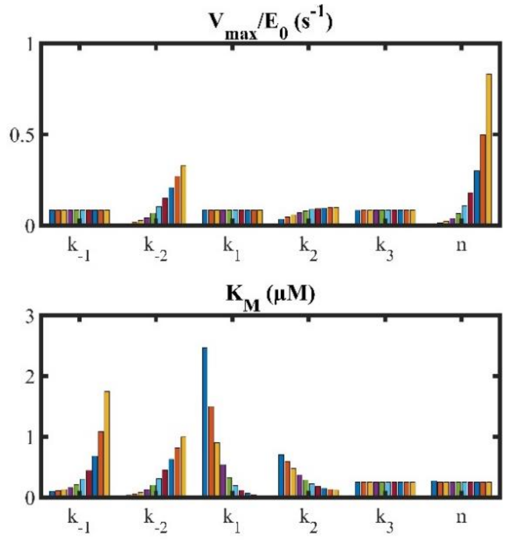

Supplementary figure 3. *Apparent Michaelis-Menten parameters derived from figure S2. All curves in supplementary figure 2 were fitted to a Michaelis-Menten equation and the derived parameters (K_M and V_{max}/E_0) obtained from the non-linear regression analysis are shown as bar plot. For each of the 6 parameters there are 10 bars, representing the magnitude of K_M (lower panel) and V_{max}/E_0 (upper panel) as the parameter was changed.*

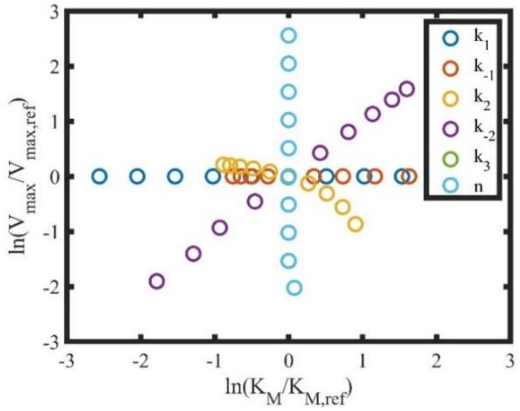

Supplementary figure 4. *Correlation plot between V_{max} and K_M. The values shown in supplementary figure 3 were plotted for each of the 6 parameters, shown in the legend. To illustrate the connection to the linear free energy relationship (LFER) reported in the main manuscript, the parameters were normalized and log scaled. We normalized all parameters with the K_M and V_{max} values, obtained from a simulation using the parameters from supplementary table 1.*

As seen from supplementary figure 3, only the processivity (*n*) and the dethreading had significant impact on the apparent k_{cat}. This result is in accordance with another model for processive cellulase (18) and experimental data (19), which show that TrCel7A and possibly other cellulases have a low turnover number due to a low dissociation rate. From supplementary figure 3 it may also be seen that the apparent K_M is more complicated to interpret. However, only the change in k_{-2} (rate constant for the dethreading) gave rise to a linear scaling between the apparent K_M and the apparent k_{cat}. This point is illustrated in supplementary figure 4 where the normalized parameters are plotted on a logarithmic scale. Supplementary figure 4 serves to show that although the apparent K_M may be influenced by several of the rate-constants in supplementary scheme 1, only change in the dethreading give scaling as the observed scaling described in the main text.

Supplementary Note 2 | Justification of K_M as an affinity parameter

Since the MM-model used to analyse the steady-state kinetics is a simplification of the system, the MM-parameters will be composite parameters. In the main text, we used the apparent K_M as a descriptor for the enzyme-substrate affinity and k_{cat} as a descriptor for the dissociation step (the rate constant for the unbinding). To support the kinetic analysis and interpretation of the MM-parameters, we measured the binding kinetics of a selected group of cellulases from fig. 2 of the main text. We have recently developed such method for cellulases (4) based on intrinsic fluorescence and we selected three wild type (WT) cellulases for this new analysis. The three WT cellulases were TrCel6A, TrCel7B and HiCel45A of the main manuscript. Together with already published data for TrCel7A and four variants of TrCel7A (4) these enzymes cover a broad spectrum of affinity and structural diversity of cellulases. In the additional experiments conducted here we made real-time measurements of the change in fluorescence upon complexation with different loads of amorphous cellulose (RAC), as described in detail elsewhere (4). The experiments could unfortunately not be done using Avicel as substrate since the large particle size gave raise to excessive light scattering.

The complex reaction shown in supplementary scheme 1 can be significantly simplified if the concentration of threaded complex (ES_{thread}) can be measured experimentally. Is such case, the reaction can be represented by a simple binding scheme (supplementary scheme 2) where the rate-constants k_{on} and k_{off} govern the threading and dethreading step respectively.

$$E + S \underset{k_{off}}{\overset{k_{on}}{\rightleftarrows}} ES_{thread} \quad \text{(Supplementary scheme 2)}$$

The raw-data (see supplementary figure 5A) was fitted to a single exponential function (supplementary eq. 7) with a constant term representing the steady-state fluorescence signal (F_{ss}).

$$F(t) = F_{ss} + Ae^{-t/b} \quad \text{(Supplementary eq. 7)}$$

The steady-state signal (F_{ss}) was fitted to a single-site binding isotherm (supplementary eq. 8) to determine the dissociation constant (K_D) and the maximal fluorescence signal (F_{max}), which is obtained when all enzymes are threaded.

$$F_{ss}(S_0) = \frac{F_{\max} S_0}{S_0 + K_D} \;,\; K_D = \frac{k_{off}}{k_{on}} \quad \text{(Supplementary eq. 8)}$$

The threading rate (v_{on}) can now be estimated from the initial slope of the curves in figure S5A. We estimated this rate from the derivate of supplementary eq. 7 solved for t=0 ($F'(t=0) = -\frac{A}{b}e^{-0/b} = -\frac{A}{b}$).

Since $ES \rightarrow E_0$ for t→0 we can write the on-rate as shown in supplementary equation 9

$$v_{on} = E_0 S_0 k_{on} = E_0 \frac{-A}{F_{\max} b} \quad \text{(Supplementary eq. 9)}$$

Where E_0 and S_0 are the initial concentration of enzyme and substrate and A, b and F_{max} are fitting parameters from respectively supplementary equation 7 and 8.

Supplementary equation 7 was fitted to the raw data shown in supplementary figure 5 to obtain the parameters A, b and F_{ss}. The latter parameter was plotted against the substrate load and fitted to equation supplementary 8 (supplementary figure 5B). Using supplementary equation 9 and the derived parameters A, b and F_{max} we could plot the on rate (v_{on}) as a function of substrate load (S_0) for the three cellulases (supplementary figure 5C).

The bi-molecular rate-constant k_{on} was derived from the slope of the curves in supplementary figure 5C and k_{off} was derived from the K_D values ($k_{off} = K_D * k_{on}$). The rate-constants for the three enzymes, as well as recently published data are given in supplementary table 2.

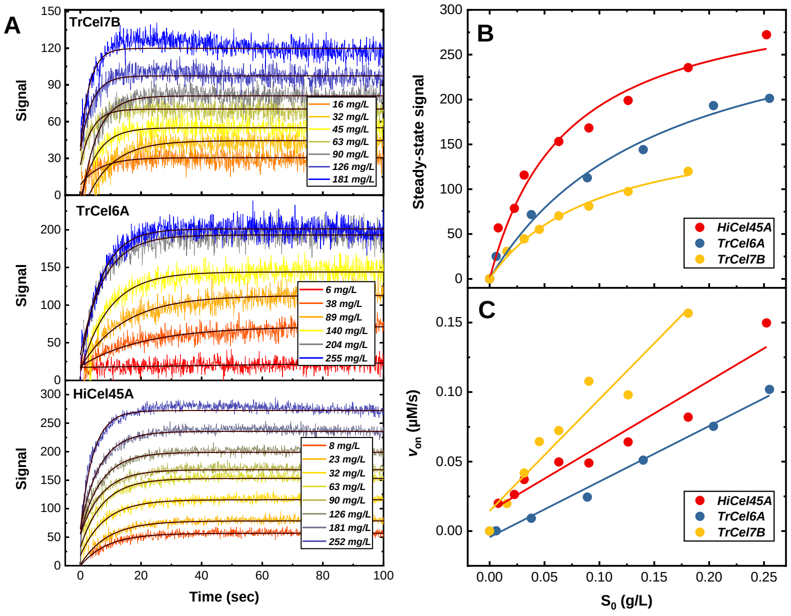

***Supplementary figure 5**. A) Real time fluorescence data for the complexation of RAC and respectively TrCel7B, TrCel6A and HiCel45A (250 nM enzyme). The ordinates show fluorescence emission in arbitrary units, and is related to the fraction of enzyme molecules in the threaded (ES_{thread}) complex. Black lines represent best fit to the exponential function supplementary eq. 7. The final RAC load in each sample is given in the legends. B) Steady-state fluorescence emission at different RAC loads. Solid lines represent best fit to supplementary eq. 8. C) Rate of threading (v_{on}) for different RAC load. The threading rate was calculated using supplementary eq. 9. The parameters in supplementary equation 9 were obtained from the non-linear regression analysis shown in panel A and B.*

***Supplementary table 2**.Parameters derived from the data shown in supplementary figure 5 and published data from Røjel et al. (4).*

Enzyme	***Mode of action***	***CBM***	k_{on} (L/g s^{-1})	$k_{off} \cdot 10^{-3}$ (s^{-1})	K_D (mg/L)	***Reference***
TrCel7B	EG	+	0.80 ± 0.09	79.3 ± 14.4	99.2 ± 14.1	
HiCel45A	EG	+	0.47 ± 0.05	32.8 ± 7.5	70.0 ± 14.2	Sup. Fig. 5
TrCel6A	CBH	+	0.40 ± 0.02	43.4 ± 16.4	105.6 ± 39.6	
TrCel7A	CBH	+	0.59 ± 0.01	4.8 ± 1.3	8.2 ± 2.2	
$TrCel7A_{CD}$	CBH	-	0.33 ± 0.02	5.9 ± 2.8	18.2 ± 8.3	
$TrCel7A_{W38A}$	CBH	+	0.41 ± 0.06	16.6 ± 8.1	40 ± 19.2	(4)
$TrCel7A_{W40A}$	CBH	+	0.39 ± 0.01	9.4 ± 3.0	23.8 ± 7.4	
$TrCel7A_{W376A}$	CBH	+	0.43 ± 0.06	2.2 ± 0.7	5.2 ± 1.5	

To further corroborate the analysis used in the main article, we calculated the change in binding free energy and activation free energy for the complexation as in the main text (eq. 2 and eq. 3, main text), where K_M and k_{cat} are substituted with respectively the dissociation constant for the complexation (K_D) and the rate-constant for either the threading (k_{on}) or dethreading (k_{off}).

$$\Delta\Delta G_B = RT \ln\left(\frac{K_D}{K_{D,ref}}\right) \text{ (supplementary eq. 10)}$$

$$\Delta\Delta G^{\ddagger} = -RT \ln\left(\frac{k}{k_{,ref}}\right) \text{ (supplementary eq. 11)}$$

Similar to the main manuscript, we have in supplementary eq. 10 and 11 normalized all energies with respect to TrCel6A, a GH6 cellobiohydrolase from *Trichoderma reesei*. The scaling between the activation energy and binding energy is shown in supplementary figure 6 for all enzymes listed in supplementary table 2. As seen from the figure, the change in the activation energy for the dethreading step ($\Delta\Delta G^{\ddagger}_{off}$) scales with the change in binding free energy($\Delta\Delta G^{0}_{B}$) with a slope of -1.05 ± 0.10. This observation is similar to the finding in the main manuscript where the binding energies and activation energies derived from the Michalis-Menten parameters scaled with a slope of – 0.74 ± 0.02. This independent set of experiments supports our interpretation of the apparent MM-parameters and justify the use of eq. 2 and 3 of the main text to derive binding energies and activation energies.

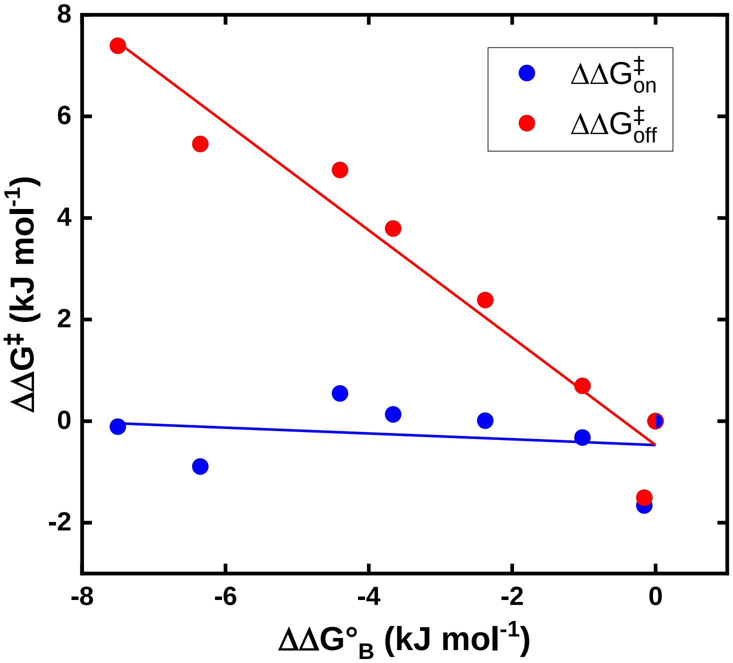

Supplementary figure 6. *Correlation plot of change in the binding free energy (ΔΔG°$_B$, Eq. S10) and activation free energy (ΔΔG‡, Eq. S11) for all enzymes shown in table S2. The blue and red symbols respectively show how the activation free energy for the threading (ΔΔG$^{\ddagger}_{on}$) and dethreading (ΔΔG$^{\ddagger}_{off}$) changes with binding (ΔΔG°$_B$). Solid lines derive from the linear regression to the experimental data.*

Supplementary Note 3 | Derivation of Optimal Michealis-Menten Constant (K_M^{opt})

Equation 6 in the main manuscript (here supplementary Eq. 12) show how K_M determines the initial rate.

$$v = \frac{E_0 A K_m{}^a S_0}{S_0 + K_M} \qquad \text{(Supplementary Eq. 12)}$$

The optimal K_M can be found by solving the equation $\frac{dV}{dK_M} = 0$ for K_M. Using the quotient rule $\frac{d}{dx}\left(\frac{u}{v}\right) = \frac{v\frac{du}{dx} - u\frac{dv}{dx}}{v^2}$ the derivative of supplementary Eq. 12 can be calculated

$$\frac{dV / E_0}{dK_M} = \frac{S_0 A K_m{}^{a-1}(a(S_0 + K_M) - K_M)}{\left(S_0 + K_M\right)^2} = 0$$

Solving for K_M give

$$K_M^{opt} = S_0 \frac{a}{1-a} \qquad \text{(Supplementary eq. 13)}$$

Using supplementary eq. 12 and 13 we simulated volcano-curves and their Sabatier optima for 50 substrate loads ranging from 1 to 100 g/L (See supplementary figure 7).

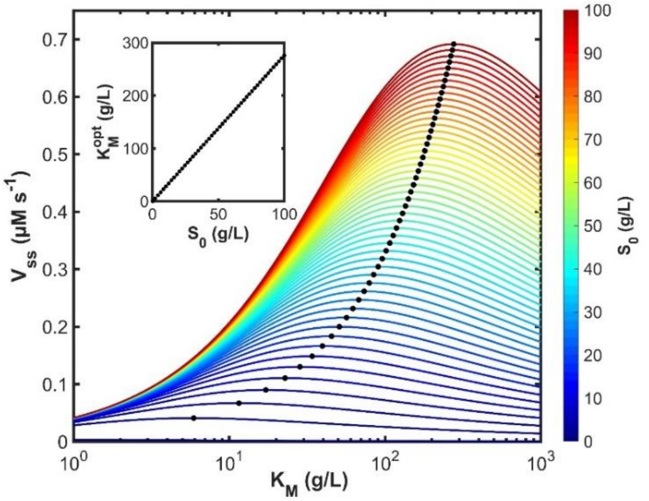

Supplementary figure 7. *Simulated volcano curves for 50 different substrate load ranging from 1 g/L to 100 g/L. The black circles indicate the optimal affinity (* K_M^{opt} *) and the insert show how* K_M^{opt} *scale with the substrate load (*S_0*). The curves were simulated with supplementary eq. 12 and* K_M^{opt} *was predicted using supplementary eq. 13.*

Supplementary Note 4 | Insoluble reducing ends measurements

All calculated rates in the main manuscript are based on the amount of soluble products. Hence, the endolytic activity of endoglucanases (EGs) is not accounted for in the analysis. This could lead to an underestimation of the EGs activities if they have a significant production of insoluble products (e.g. insoluble cellooligosacchaerides or new chain ends). In order to quantify possible systematic underestimations of the activities of the EGs, we measured the amount of insoluble reducing ends for a representative group of enzymes. The group included EGs from all of the investigated GH families and two CBH enzymes from family 6 and 7. In general, the results showed that the insoluble activity was small compared to the soluble activity (see table S3). Only TrCel12A had a significant insoluble activity (46%), which may explain why this enzyme appeared at the edge of the 95% prediction band in Fig. 2 (main text). A brief description of the materials and methods used for these measurements is described below.

The results in supplementary table 3 were estimated by adapting the method described in Silveira et al. (20) and by using Bicinchoninic acid (BCA) in presence of $CuSO_4$. The reactions were carried out in 2 mL microcentrifuge tubes (Sarstedt, Numbrecht, Germany). Enzymes and washed Avicel (Avicel PH-101, Sigma-Aldrich, Steinheim, Germany) were mixed in 300 µL to a final enzyme concentration of 0.1 µM and Avicel load of 40 g/L. The reactions were incubated for 1h at 25 °C in thermomixers (Eppendorf, Hamburg, Germany) equipped with ThermoTop and operating at 1100 rpm. The reactions were then stopped by transfer on ice where the rest of the procedure followed. From the reactions, 25 µL were retrieved and standard buffer was added to a final volume of 500 µL. These samples were used to quantify the total reducing ends (TRE). The initial reactions were then centrifuged for 3 min at 14100 rcf. From these, 25 µL were retrieved and standard buffer was added to a final volume of 500 µL. The samples were centrifuged again as described before. These samples were used to measure the amount of soluble reducing ends (SRE). The amount of insoluble reducing ends where calculated by subtracting SRE from the TRE. A volume of 150 µL was retrieved from both TRE and SRE samples, and 150 µL of Bicinchoninic acid (BCA) reagent was added. BCA reagent was prepared by mixing equal volumes of solution A (0.46 M sodium carbonate, 0.26 M sodium bicarbonate and 4.5 mM Bicinchoninic acid disodium salt hydrate in deionized water) and solution B (4.5 mM Copper(II) sulfate pentahydrate and 10 mM L-serine in deionized water). The samples were briefly vortexed and incubated in thermomixers (Eppendorf, Hamburg, Germany) for 30 min at 75°C and 1100 rpm. The reactions were then cooled for 10 min at 4 °C, vortexed again, and centrifuged for 3 min as described above. Finally, 100 µL of supernatant were transferred to a 96 well microtiter plate (655101, Greiner Bio-One, Germany) and the absorbance at 560 nm was measured using a spectrophotometer (Spectramax i3, Molceular Devices, Wals, Austria). A 6 point calibration curve of cellobiose (3.9-125 µM) was included in all measurements. Results from the above analysis are reported in table S3 for a selected group of cellulases. The group was selected so that all types of endoglucanases and cellobiohydrolases were covered.

Supplementary table 3. *Concentration of total reducing end (TRE), soluble reducing ends (SRU) and insoluble reducing ends (IRE) for four endoglucanases (EG) and two cellobiohydrolases (CBH) representing all GH families charecterised in the main text.*

Enzyme	*Mode of action*	*TRE (µM)*	*SRE (µM)*	*IRE (µM)*	*IRE/TRE *100*
TrCel5A	EG	169 ±30	156 ±3	13 ±30	8
TrCel12A	EG	145 ±56	78 ±1.5	67 ±56	46
HiCel45A	EG	131 ±9	124.5 ±0.4	6 ±9	5
TrCel7B	EG	220 ±28	211 ±2	9 ±28	4
TrCel7A	CBH	35 ±15	36 ±6	n.d.	n.d.
TrCel6A	CBH	98 ±63	103 ±4	n.d.	n.d.

Supplementary Note 5 | Illustration of the MD method used to compute ligand binding energies

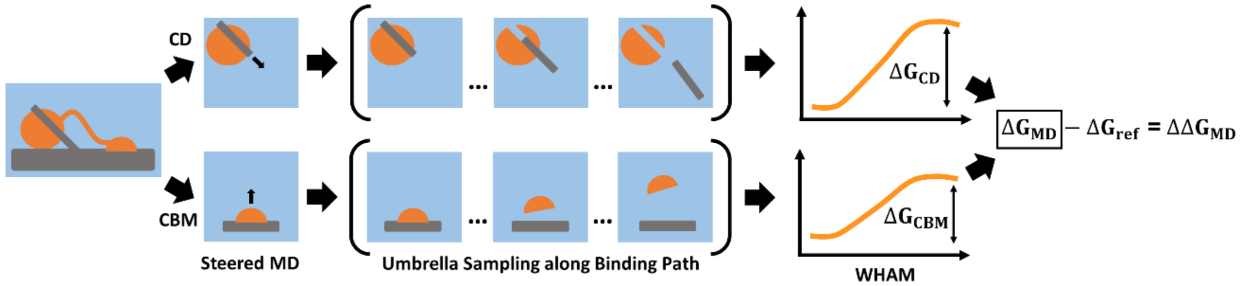

Supplementary figure 8. *Illustration of the workflow used to compute the binding energies in this study. The binding energies were derived from steered molecular dynamics (MD) simulation with umbrella sampling along the binding path. This was done in parallel for the catalytic domain and the carbohydrate-binding module (if present) and the simulations were analyzed using weighted histogram analysis method. In order to compare with the experimental result the obtained absolute binding energies were normalized with TrCel6A (ΔG_{ref}) to obtain the change in binding free energies ($\Delta\Delta G_{MD}$) which could be compared to the experimental derived energies $\Delta\Delta G_{exp}$.*

Supplementary Note 6 | Correlation plot of change in the free energy of binding and activation for all investigated enzymes

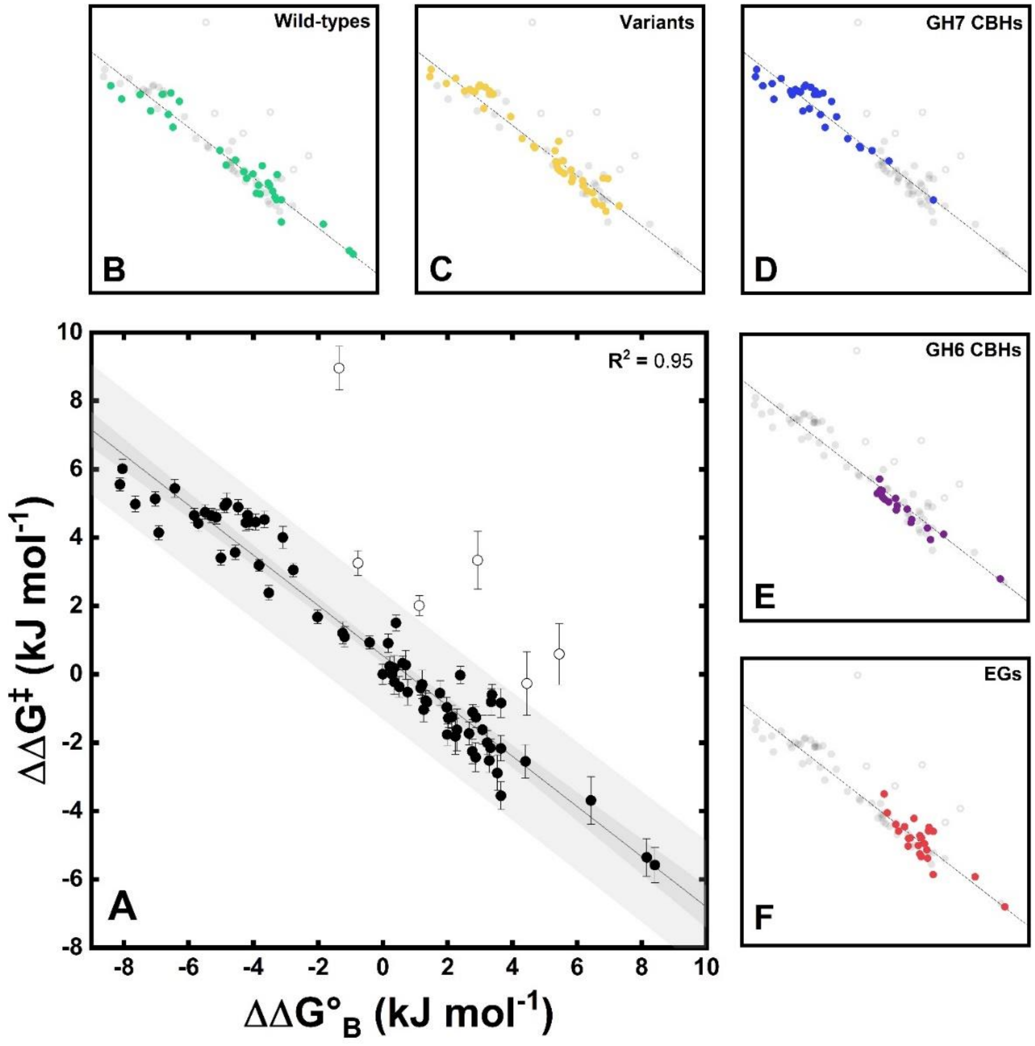

Supplementary figure 9. *Correlation plot of change in the binding free energy ($\Delta\Delta G^{o}{}_{B}$) and activation free energy ($\Delta\Delta G^{\ddagger}$) for all investigated enzymes (A). The smaller panels highlight data for different classes of enzymes. These are wild type cellulases (B), variants (C), cellobiohydrolases from GH7 (D), cellobiohydrolases from GH6 (E), and endoglucanases from family GH7, GH12, and GH45 (F). The solid line in all plots derives from the linear regression to all the experimental data of the main panel (A) excluding the outliers (open symbols) identified as explained in the main text. Bands shown in panel A are 95% confidence band (dark gray) and 95% prediction band (light gray). All free energies are calculated using the kinetic parameters in supplementary table 4 and Eq. 2 and Eq. 3 (main text).*

Supplementary table 4. Wild-type and variant enzymes properties. "O", catalytic domain; "o", CBM1; "---", linker. Blue indicates mutations in the catalytic domain, purple in the CBM, and yellow in the linker. **Bold** indicates chimeric enzymes where a CBM were added or substituted.

Organism	GH	Mechanism	WT or Variant	Modularity	Mutations	Public sequence[1]	Ref.[2]	k_{cat} (s^{-1})	K_M (g/L)	$(k_{cat} / K_M)*10^3$ ($s^{-1}gL^{-1}$)
**Cellobiohydrolases**										
Trichoderma reesei	7	Retaining	WT	O---o	N/A	P62694 (U)	(19)	0.095 ± 0.003	2.6 ± 0.3	35.8 ±2.6
Penicillium chrysogenum	7	Retaining	WT	O---o	N/A	Q5S1P9 (U)		0.388 ± 0.006	22.4 ± 1.0	17.3 ±0.5
Talaromyces leycettanus	7	Retaining	WT	O---o	N/A	S6EXC0 (U)		0.076 ± 0.003	1.2 ± 0.1	62.6 ±4.9
Rasamsonia emersonii	7	Retaining	WT	O	N/A	Q8TFL9 (U)	(21)	0.091 ± 0.003	6.0 ± 0.7	15 ±1.1
Rasamsonia byssoclamydoides	7	Retaining	WT	O	N/A	S6EJQ7 (U)		0.112 ± 0.008	7.6 ± 1.3	14.7 ±1.5
Neosartorya fischeri	7	Retaining	WT	O	N/A	A1DMA5 (U)		1.354 ± 0.119	115.1 ± 15.7	11.8 ±0.6
Neosartorya fischeri	7	Retaining	WT	O---o	N/A	A1DAP8 (U)		0.106 ± 0.003	1.6 ± 0.2	65.4 ±4.7
Aspergillus terreus	7	Retaining	WT	O---o	N/A	Q0CMT2 (U)		0.216 ± 0.006	6.4 ± 0.6	33.9 ±2.5
Colletotrichum graminicola	7	Retaining	WT	O	N/A	E3Q986 (U)		0.094 ± 0.003	4.8 ± 0.5	19.6 ±1.6
Phanerochaete chrysosporium	7	Retaining	WT	O---o	N/A	P13860 (U)		0.143 ± 0.004	3.5 ± 0.3	40.6 ±2.4
Aspergillus aculeatus	7	Retaining	WT	O	N/A	A0A1L9X3D1 (U)		*0.251 ± 0.014	*41.5 ± 5.2	*6.0 ±0.4
Trichoderma reesei	6	Inverting	WT	o---O	N/A	P07987 (U)	(22)	0.564 ± 0.034	26.4 ± 4.0	21.4 ±1.9
Lentinus sajor-caju	6	Inverting	WT	o---O	N/A	Q96TP4 (U)	(22)	1.345 ± 0.060	101.0 ± 7.5	13.3 ±0.4
Aspergillus terreus	6	Inverting	WT	O	N/A	Q0D1J1 (U)	(22)	4.900 ± 0.785	708.3 ± 127.1	6.9 ±0.1
Colletotrichum graminicola	6	Inverting	WT	o---O	N/A	E3Q540 (U)	(22)	1.172 ± 0.178	65.2 ± 19.0	18 ±2.5
Colletotrichum graminicola	6	Inverting	WT	O	N/A	E3Q986 (U)	(22)	U/D	U/D	9.4 ±0.1
Trichoderma reesei	7	Retaining	Variant	O	Deletion of linker and CBM	N/A	(19)	0.165 ± 0.003	8.7 ± 0.6	19.1 ±0.9
Trichoderma reesei	7	Retaining	Variant	O---o---**o**	CBM and linker from TrCel7A	N/A		0.087 ± 0.002	2.5 ± 0.2	34.4 ±2
Trichoderma reesei	7	Retaining	Variant	O---o	W38A	N/A	(19)	0.506 ± 0.056	35.1 ± 9.0	14.4 ±2.1
Trichoderma reesei	7	Retaining	Variant	O---o	W40A	N/A	(4)	0.134 ± 0.003	4.2 ± 0.4	31.9 ±2.3
Trichoderma reesei	7	Retaining	Variant	O---o	T246C/Y371C	N/A	(23)	0.094 ± 0.003	5.4 ± 0.6	17.3 ±1.2
Trichoderma reesei	7	Retaining	Variant	O---o	Δ(W192-G205)	N/A	(24)	0.347 ± 0.025	16.0 ± 3.3	21.7 ±3
Trichoderma reesei	7	Retaining	Variant	O---o	Δ(E193-G205)	N/A	(24)	0.363 ± 0.022	16.4 ± 2.9	22.1 ±2.6
Trichoderma reesei	7	Retaining	Variant	O---o	Δ(S196-T201)	N/A	(24)	0.287 ± 0.007	11.7 ± 0.9	24.5 ±1.3
Trichoderma reesei	7	Retaining	Variant	O-o	Δ(G439-G444)	N/A		0.071 ± 0.002	1.6 ± 0.2	45.9 ±3.3
Rasamsonia emersonii	7	Retaining	Variant	O**---o**	CBM and linker from *T. reesei* Cel7A	N/A	(21)	0.060 ± 0.001	1.0 ± 0.1	60 ±5
Rasamsonia emersonii	7	Retaining	Variant	O**---o**	CBM and linker from *T. reesei* Cel7A + Y470W	N/A		0.050 ± 0.003	1.0 ± 0.2	48.4 ±6.2
Rasamsonia emersonii	7	Retaining	Variant	O**---o**	CBM and linker from *T. reesei* Cel7A + Y478W	N/A		0.093 ± 0.002	5.0 ± 0.4	18.7 ±1.2
Rasamsonia emersonii	7	Retaining	Variant	O**---o**	CBM and linker from *T. reesei* Cel7A + Y478W/Y497W	N/A		0.077 ± 0.001	3.7 ± 0.2	20.9 ±1
Rasamsonia emersonii	7	Retaining	Variant	O**---o**	CBM and linker from *T. reesei* Cel7A + Y496W/Y497W	N/A		0.063 ± 0.003	2.0 ± 0.3	31.7 ±2.7
Rasamsonia emersonii	7	Retaining	Variant	O**---o**	CBM and linker from *T. reesei* Cel7A + Y470W/Y478W	N/A		0.088 ± 0.002	3.3 ± 0.2	26.6 ±1.4
Rasamsonia emersonii	7	Retaining	Variant	O**---o**	CBM and linker from *T. reesei* Cel7A + Y478W/Y496W	N/A		0.092 ± 0.003	4.9 ± 0.6	18.7 ±1.6
Rasamsonia emersonii	7	Retaining	Variant	O**---o**	CBM and linker from *T. reesei* Cel7A + Y470W/Y496W	N/A		0.086 ± 0.002	4.9 ± 0.3	17.6 ±0.7
Rasamsonia emersonii	7	Retaining	Variant	O**---o**	CBM and linker from *T. reesei* Cel7A + Y470W/Y496W/Y497W	N/A		0.083 ± 0.002	2.9 ± 0.2	28.8 ±1.6
Rasamsonia emersonii	7	Retaining	Variant	O**---o**	CBM and linker from *T. reesei* Cel7A + Y478W/Y496W/Y497W	N/A		0.087 ± 0.002	3.1 ± 0.3	27.8 ±1.6
Rasamsonia emersonii	7	Retaining	Variant	O**---o**	CBM and linker from *T. reesei* Cel7A + Y470W/Y478W/Y496W/Y497W	N/A		0.075 ± 0.004	3.8 ± 0.7	19.7 ±2.3
Rasamsonia emersonii	7	Retaining	Variant	O**---o**	CBM and linker from *T. reesei* Cel7A + Y470W/Y478W/Y496W	N/A		0.078 ± 0.002	4.4 ± 0.4	18 ±1.2
Trichoderma reesei	6	Inverting	Variant	O	Deletion of linker and CBM	N/A	(25)	0.834 ± 0.047	58.7 ± 6.5	14.2 ±0.8
Trichoderma reesei	6	Inverting	Variant	o---O	Y103A	N/A		0.618 ± 0.054	30.5 ± 6.4	20.3 ±2.5
Trichoderma reesei	6	Inverting	Variant	o---O	N305A	N/A		0.856 ± 0.071	44.0 ± 7.7	19.5 ±1.8
Trichoderma reesei	6	Inverting	Variant	o---O	S106A	N/A		0.697 ± 0.064	36.0 ± 7.6	19.4 ±2.3
Trichoderma reesei	6	Inverting	Variant	o---O	R410A	N/A		0.524 ± 0.044	30.3 ±6.1	17.3 ±2
Trichoderma reesei	6	Inverting	Variant	o---O	G365D/D366N	N/A		0.637 ± 0.069	43.1 ± 10.0	14.8 ±1.8
Trichoderma reesei	6	Inverting	Variant	o---O	W367F	N/A		1.087 ± 0.204	66.6 ± 23.8	16.3 ±2.8
Trichoderma reesei	6	Inverting	Variant	o---O	W269A	N/A		1.578 ± 0.216	156.4 ± 30.7	10.1 ±0.6
Trichoderma reesei	6	Inverting	Variant	o---O	W272A	N/A		1.809 ± 0.268	110.0 ± 26.5	16.4 ±1.5
Trichoderma reesei	6	Inverting	Variant	o---O	K395T	N/A		*0.015 ± 0.003	*15.3 ± 8.9	*1.0 ±0.4
Trichoderma reesei	6	Inverting	Variant	o---O	W269A/W272A	N/A		*0.629 ± 0.198	*159.0 ± 74.1	*4.0 ±0.6
Trichoderma reesei	6	Inverting	Variant	**o**---O	CBM from *P. anserina* GH6	N/A	(26)	0.514 ± 0.028	28.8 ± 3.8	17.8 ±1.4
Trichoderma reesei	6	Inverting	Variant	**o**---O	CBM from *N. frontalis* GH6	N/A	(26)	0.564 ± 0.028	29.6 ± 3.5	19.1 ±1.3
Trichoderma reesei	6	Inverting	Variant	**o**---O	CBM from *S. indica* GH6	N/A	(26)	0.391 ± 0.020	28.2 ± 3.6	13.9 ±1.0
Trichoderma reesei	6	Inverting	Variant	**o**---O	CBM from *C. cinerea* GH6	N/A	(26)	0.770 ± 0.042	45.1 ± 5.1	17.1 ±1.0
Trichoderma reesei	6	Inverting	Variant	**o**---O	CBM from *V. volvacea* GH6	N/A	(26)	0.654 ± 0.040	32.4 ± 4.7	20.2 ±1.7
**Endoglucanases**										
Trichoderma reesei	7	Retaining	WT	O---o	N/A	P07981 (U)	(24)	2.365 ± 0.243	115.2 ± 18.4	20.5 ±1.2
Chaetomium virescens	7	Retaining	WT	O	N/A	BDB18317 (GS)		5.360 ± 0.800	782.9± 128.1	6.8 ±0.1
Aspergillus terreus	7	Retaining	WT	O	N/A	Q0CC84 (U)		U/D	U/D	5.0 ±0.1
Trichoderma reesei	5	Retaining	WT	O---o	N/A	P07982 (U)	(12)	1.150 ± 0.077	58.9 ± 7.6	19.5 ±1.2
Thermoascus aurantiacus	5	Retaining	WT	O	N/A	Q8TG26 (U)		2.500 ± 0.553	352.9 ± 94.1	7.1 ±0.3
Penicillium brasilianum	5	Retaining	WT	O---o	N/A	B8Q961 (U)		0.785 ± 0.038	45.7 ± 4.6	17.2 ±0.9
Neosartorya fischeri	5	Retaining	WT	O---o	N/A	A1DNK9 (U)		0.935 ± 0.070	62.5 ± 8.8	15 ±1
Gloephyllum trabeum	5	Retaining	WT	O	N/A	D7REW1 (U)		0.705 ± 0.061	53.8 ± 9.2	13.1 ±1.1
Chaetomium virescens	5	Retaining	WT	O	N/A	BDB1821(GS)		1.086 ± 0.119	91.4 ± 16.7	11.9 ±0.9
Colletotrichum graminicola	5	Retaining	WT	o---O	N/A	E3QRW7 (U)		0.938 ± 0.057	84.2 ± 8.7	11.1 ±0.5
Aspergillus aculeatus	5	Retaining	WT	O	N/A	A0A1L9WYV6 (U)			Activity too low to measure	
Humicola insolens	45	Inverting	WT	O---o	N/A	P43316 (U)		1.265 ± 0.102	97.0 ± 12.9	13 ±0.7
Humicola hyalothermophila	45	Inverting	WT	O---o	N/A	BBJ30934 (GS)		0.884 ± 0.027	80.8 ± 4.3	10.9 ±0.2
Acremonium furcatum	45	Inverting	WT	O---o	N/A	AAY00847.1 (GB)		0.664 ± 0.016	42.3 ± 2.1	15.7 ±0.4
Lectera colletotrichoides	45	Inverting	WT	O---o	N/A	AAY00854.1 (GB)		0.495 ± 0.010	33.8 ± 1.6	14.6 ±0.4
Rhizomucor pusillus	45	Inverting	WT	O---o	N/A	BBK79186 (GS)		0.571 ± 0.024	69.2 ± 5.4	8.3 ±0.3
Melanocarpus albomyces	45	Inverting	WT	O	N/A	Q8J0K8 (U)		*0.152 ± 0.013	*19.4 ± 4.7	*7.8 ±1.2
Trichoderma reesei	12	Retaining	WT	O	N/A	G0RRG8 (U)	(27)	0.718 ± 0.044	102.9 ± 10.2	7.0 ±0.3
Aspergillus aculeatus	12	Retaining	WT	O	N/A	A0A1L9WFV2 (U)			Activity too low to measure	
Chaetomium virescens	12	Retaining	WT	O	N/A	BDB18192 (GS)		*0.147 ± 0.041	*86.0 ± 40.8	*1.7 ±0.3
Trichoderma reesei	7	Retaining	Variant	O---o	D76R	N/A		0.947 ± 0.052	59.7 ± 6.2	15.9 ±0.8
Trichoderma reesei	7	Retaining	Variant	O---o	D62A/E63A	N/A		0.308 ± 0.010	31.1 ± 2.4	9.9 ±0.4
Trichoderma reesei	7	Retaining	Variant	O---o	D115A	N/A		1.502 ± 0.169	84.0 ± 16.7	17.9 ±1.5
Trichoderma reesei	7	Retaining	Variant	O---o	D151A, E152A	N/A		1.135 ± 0.084	77.3 ± 10.4	14.7 ±0.9
Trichoderma reesei	7	Retaining	Variant	O---o	D366P	N/A		1.404 ± 0.120	80.6 ± 11.9	17.4 ±1.1
Trichoderma reesei	7	Retaining	Variant	O---o	K122T	N/A		1.564 ± 0.130	99.5 ± 13.4	15.7 ±0.8
Trichoderma reesei	12	Retaining	Variant	O	Y111W	N/A		0.792 ± 0.090	115.4 ± 21.0	6.9 ±0.5
Trichoderma reesei	12	Retaining	Variant	O	S63I	N/A		0.783 ± 0.076	101.9 ± 16.4	7.7 ±0.5
Trichoderma reesei	12	Retaining	Variant	O	W7G	N/A		*0.445 ± 0.134	*238.0 ± 95.5	*1.9 ±0.2
Trichoderma reesei	12	Retaining	Variant	O	W22A	N/A			Activity too low to measure	

[1]UniProt (U), Genbank (GB) or GENESEQ (GS).

[2]Enzyme was constructed, expressed and purified as described in reference.

*Identified as outliers in Fig. 2 in the main manuscript.

N/A = not applicable, **U/D** = unable to determine. Affinity too low to resolve K_M and k_{cat} from non-linear regression to Eq. 1 (main text). Only the ratio k_{cat}/K_M could be determined.

Supplementary table 5. List of primers.

Organism	GH	Mechanism	WT or Variant	Modularity	Mutations	*Forward*	Reverse
**Cellobiohydrolases**							
Trichoderma reesei	7	Retaining	Variant	O	Deletion of linker and CBM	ACACAACTGGGGATCCACCATGTATC<br>GTAAGCTCGCAGTCATCTCC	CTAGATCTCGAGAAGCTTAGTTTCCACCGGAGG<br>GGTTGC
Trichoderma reesei	7	Retaining	Variant	O---o---**o**	CBM and linker from TrCel7A		§
Trichoderma reesei	7	Retaining	Variant	O---o	W38A	CGGTGGTCATCGACGCCAACGCAAG<br>GTGGACGCATGCAACCAAC	GTTGGCGTCGATGACCACCGAGCCAGTCTGC
Trichoderma reesei	7	Retaining	Variant	O---o	W40A	GTCATCGACGCCAACTGGAGGGCAAC<br>GCATGCAACCAACTCCTCC	CCTCCAGTTGGCGTCGATGACCACCGAGCCA
Trichoderma reesei	7	Retaining	Variant	O---o	T246C/Y371C	AAGGAGACGGCTGTGGCGGATGTTA<br>CTCCGATAACCGTTACGGTG<br>CATGTCCTTGTGGGACGACTACTGTG<br>CCAACATGCTCTGGCTCGAC	TCCGCCACAGCCGTCTCCTTCACAAATCTCCT<br>GTAGTCGTCCCACAAGGACATGACCAACACCATT
Trichoderma reesei	7	Retaining	Variant	O---o	Δ(W192-G205)	GCCAGGCAAACGTCGAAGGCCATGG<br>CTCCTGTTGTTCGGAAATG	GCCTTCGACGTTTGCCTGGCCGTTGATGAACT
Trichoderma reesei	7	Retaining	Variant	O---o	Δ(E193-G205)	CAGGCAAACGTCGAAGGCTGGCATG<br>GCTCCTGTTGTTCGGAAATG	CCAGCCTTCGACGTTTGCCTGGCCGTTGATGA
Trichoderma reesei	7	Retaining	Variant	O---o	Δ(S196-T201)	TCGAAGGCTGGGAACCCTCGGGCATT<br>GGAGGCCATGGCTCC	CGAGGGTTCCCAGCCTTCGACGTTTGCCTGG
Trichoderma reesei	7	Retaining	Variant	O-o	Δ(G439-G444)	CCTCCGGTGGAAACCCTCCTACAACT<br>ACAACACGACGGCCTG	AGGAGGGTTTCCACCGGAGGGGTTGCCGGT
Rasamsonia emersonii	7	Retaining	Variant	O---**o**	CBM and linker from *T. reesei* Cel7A		§
Rasamsonia emersonii	7	Retaining	Variant	O---**o**	CBM and linker from *T. reesei* Cel7A<br>+ Y470W	CTGGACCGACCCAGTCCCACTGGGGA<br>CAGTGTGGAGGCATCGG	GTGGGACTGGGTCGGTCCAGGGGACGAACC
Rasamsonia emersonii	7	Retaining	Variant	O---**o**	CBM and linker from *T. reesei* Cel7A<br>+ Y478W	GGACAGTGTGGAGGCATCGGTTGGT<br>CCGGTCCGACCGTCTGTGC	ACCGATGCCTCCACACTGTCCGTAGTGGGACT
Rasamsonia emersonii	7	Retaining	Variant	O---**o**	CBM and linker from *T. reesei* Cel7A<br>+ Y478W/Y497W	GGACAGTGTGGAGGCATCGGTTGGT<br>CCGGTCCGACCGTCTGTGCGTCCGGC<br>ACAACCTGTCAGGTCTTGAACCCTTAC<br>TGGTCGCAGTGTCTCTAATAGACGTG	ACCGATGCCTCCACACTGTCCGTAGTGGGACT
Rasamsonia emersonii	7	Retaining	Variant	O---**o**	CBM and linker from *T. reesei* Cel7A<br>+ Y496W/Y497W	AACCTGTCAGGTCTTGAACCCTTGGT<br>GGTCGCAGTGTCTCTAATAGACGTG	AGGGTTCAAGACCTGACAGGTTGTGCCGGACG
Rasamsonia emersonii	7	Retaining	Variant	O---**o**	CBM and linker from *T. reesei* Cel7A<br>+ Y470W/Y478W	CTGGACCGACCCAGTCCCACTGGGGA<br>CAGTGTGGAGGCATCGGTTGGTCCG<br>GTCCGACCGTCTGTGC	GTGGGACTGGGTCGGTCCAGGGGACGAACC
Rasamsonia emersonii	7	Retaining	Variant	O---**o**	CBM and linker from *T. reesei* Cel7A<br>+ Y478W/Y496W	GGACAGTGTGGAGGCATCGGTTGGT<br>CCGGTCCGACCGTCTGTGCGTCCGGC<br>ACAACCTGTCAGGTCTTGAACCCTTG<br>GTATTCGCAGTGTCTCTAATAGACG	ACCGATGCCTCCACACTGTCCGTAGTGGGACT
Rasamsonia emersonii	7	Retaining	Variant	O---**o**	CBM and linker from *T. reesei* Cel7A<br>+ Y470W/Y496W	AACCTGTCAGGTCTTGAACCCTTGGT<br>ATTCGCAGTGTCTCTAATAGACG	AGGGTTCAAGACCTGACAGGTTGTGCCGGACG
Rasamsonia emersonii	7	Retaining	Variant	O---**o**	CBM and linker from *T. reesei* Cel7A<br>+ Y470W/Y496W/Y497W	CTGGACCGACCCAGTCCCACTGGGGA<br>CAGTGTGGAGGCATCGG	GTGGGACTGGGTCGGTCCAGGGGACGAACC
Rasamsonia emersonii	7	Retaining	Variant	O---**o**	CBM and linker from *T. reesei* Cel7A<br>+ Y478W/Y496W/Y497W	GGACAGTGTGGAGGCATCGGTTGGT<br>CCGGTCCGACCGTCTGTGC	ACCGATGCCTCCACACTGTCCGTAGTGGGACT
Rasamsonia emersonii	7	Retaining	Variant	O---**o**	CBM and linker from *T. reesei* Cel7A<br>+ Y470W/Y478W/Y496W/Y497W	CTGGACCGACCCAGTCCCACTGGGGA<br>CAGTGTGGAGGCATCGGTTGGTCCG<br>GTCCGACCGTCTGTGC	GTGGGACTGGGTCGGTCCAGGGGACGAACC
Rasamsonia emersonii	7	Retaining	Variant	O---**o**	CBM and linker from *T. reesei* Cel7A<br>+ Y470W/Y478W/Y496W	AACCTGTCAGGTCTTGAACCCTTGGT<br>ATTCGCAGTGTCTCTAATAGACG	AGGGTTCAAGACCTGACAGGTTGTGCCGGACG
Trichoderma reesei	6	Inverting	Variant	O	Deletion of linker and CBM	TACGCTGGCCACACTCGCAGCTAGTT<br>CGGGAACCGCTACGTATTCAGGC	TTACAGGAACGATGGGTTTGCGTTTGT<br>GAG
Trichoderma reesei	6	Inverting	Variant	o---O	Y103A	CTTGGGCCAATGCAGCTTACGCCTCT<br>GAAG	CTTCAGAGGCGTAAGCTGCATTGGCCCAAG
Trichoderma reesei	6	Inverting	Variant	o---O	N305A	GGCAACCAATGTCGCCGCCTACAACG<br>GGTGGAAC	GTTCCACCCGTTGTAGGCGGCGACATTGGTTGCC
Trichoderma reesei	6	Inverting	Variant	o---O	S106A	CAATGCATATTACGCCGCTGAAGTTA<br>GCAGCCTC	GAGGCTGCTAACTTCAGCGGCGTAATATGCATTG<br>G
Trichoderma reesei	6	Inverting	Variant	o---O	R410A	CAGCAGTGCGCCAGCATTTGACTCCC<br>AC	GTGGGAGTCAAATGCTGGCGCACTGCTG
Trichoderma reesei	6	Inverting	Variant	o---O	G365D/D366N	GGACAGCAACAGTGGGACAACTGGT<br>GCAATGTGATC	GATCACATTGCACCAGTTGTCCCACTGTTGCTGT<br>CC
Trichoderma reesei	6	Inverting	Variant	o---O	W367F	CAGTGGGGAGACTTCTGCAATGTGAT<br>CG	CGATCACATTGCAGAAGTCTCCCCACTG
Trichoderma reesei	6	Inverting	Variant	o---O	W269A	CTGGCCATGCAGGAGCGCTTGGCTGG<br>CCGG	CCGGCCAGCCAAGCGCTCCTGCATGGCCAG
Trichoderma reesei	6	Inverting	Variant	o---O	W272A	GCAGGATGGCTTGGCGCCCCGGCAA<br>ACCAAGAC	GTCTTGGTTTGCCGGGGCGCCAAGCCATCCTGC
Trichoderma reesei	6	Inverting	Variant	o---O	K395T	CGTTTGTCTGGGTCACGCCAGGCGGC<br>GAGTG	CACTCGCCGCCTGGCGTGACCCAGACAAACG
Trichoderma reesei	6	Inverting	Variant	o---O	W269A/W272A	GCAGGAGCGCTTGGCGCCCCGGCAA<br>ACCAAGAC	GTCTTGGTTTGCCGGGGCGCCAAGCGCTCCTGC
Trichoderma reesei	6	Inverting	Variant	**o**---O	CBM from *P. anserina* GH6		§
Trichoderma reesei	6	Inverting	Variant	**o**---O	CBM from *N. frontalis* GH6		§
Trichoderma reesei	6	Inverting	Variant	**o**---O	CBM from *S. indica* GH6		§
Trichoderma reesei	6	Inverting	Variant	**o**---O	CBM from *C. cinerea* GH6		§
Trichoderma reesei	6	Inverting	Variant	**o**---O	CBM from *V. volvacea* GH6		§
**Endoglucanases**							
Trichoderma reesei	7	Retaining	Variant	O---o	D76R	AACTGCTTCATCGAGGGCGTCAGGTA<br>CGCCGCCTCGGGCGTCA	GACGCCCTCGATGAAGCAGTTCTTGCCACAGG
Trichoderma reesei	7	Retaining	Variant	O---o	D62A/E63A	GTCAACACCACGCTCTGCCCTGCCGC<br>CGCGACCTGTGGCAAGAACTGC	AGGGCAGAGCGTGGTGTTGACGCCGCCGTTG
Trichoderma reesei	7	Retaining	Variant	O---o	D115A	CGGCTGTATCTCCTGGACTCTGCCGG<br>TGAGTACGTGATGCTGAAGC	AGAGTCCAGGAGATACAGCCGAGGAGAGACGC
Trichoderma reesei	7	Retaining	Variant	O---o	D151A, E152A	CTCGCTCTACCTGTCTCAGATGGCCGC<br>CAACGGGGGCGCCAACCAGTA	CATCTGAGACAGGTAGAGCGAGCCGTTCTCTCC
Trichoderma reesei	7	Retaining	Variant	O---o	D366P	TTCTCCAACATCCGCTGGGGACCCATT<br>GGGTCTACTACGAACTCGAC	TCCCCAGCGGATGTTGGAGAAGACGACGTGC
Trichoderma reesei	7	Retaining	Variant	O---o	K122T	GACGGTGAGTACGTGATGCTGACCCT<br>CAACGGCCAGGAGCTGAG	CAGCATCACGTACTCACCGTCAGAGTCCAGGA
Trichoderma reesei	12	Retaining	Variant	O	Y111W	GCCAACCCGAATCATGTCACGTGGTC<br>GGGAGACTACGAACTC	CGTGACATGATTCGGGTTGGCTGCGGTGAACA
Trichoderma reesei	12	Retaining	Variant	O	S63I	CAACGTCAAGTCGTACCAGAACATCC<br>AGATTGCCATTCCCCAGAAGA	GTTCTGGTACGACTTGACGTTGTTCTGGCCGC
Trichoderma reesei	12	Retaining	Variant	O	W7G	GCCCAAACCAGCTGTGACCAGGGCGC<br>AACCTTCACTGGCAAC	CTGGTCACAGCTGGTTTGGGCCAGGGCGGC
Trichoderma reesei	12	Retaining	Variant	O	W22A	GCTACACAGTCAGCAACAACCTTGCA<br>GGAGCATCAGCCGGCTCT	AAGGTTGTTGCTGACTGTGTAGCCGTTGCCAGTG

§ gBlocks™ Gene Fragments ordered from IDT overhang of 24 bp (see main text)

Supplementary References

1. Chundawat, S. P. S., Beckham, G. T., Himmel, M. E., and Dale, B. E. (2011) Deconstruction of lignocellulosic biomass to fuels and chemicals. *Annu Rev Chem Biomol* **2**, 121-145
2. Payne, C. M., Knott, B. C., Mayes, H. B., Hansson, H., Himmel, M. E., Sandgren, M., Stahlberg, J., and Beckham, G. T. (2015) Fungal cellulases. *Chem Rev* **115**, 1308-1448
3. Cruys-Bagger, N., Alasepp, K., Andersen, M., Ottesen, J., Borch, K., and Westh, P. (2016) Rate of Threading a Cellulose Chain into the Binding Tunnel of a Cellulase. *Journal of Physical Chemistry B* **120**, 5591-5600
4. Røjel, N., Kari, J., Sørensen, T. H., Badino, S. F., Morth, J. P., Schaller, K., Cavaleiro, A. M., Borch, K., and Westh, P. (2019) Substrate binding in the processive cellulase Cel7A: Transition state of complexation and roles of conserved tryptophan residues.
5. Chundawat, S. P., Bellesia, G., Uppugundla, N., da Costa Sousa, L., Gao, D., Cheh, A. M., Agarwal, U. P., Bianchetti, C. M., Phillips, G. N., Jr., Langan, P., Balan, V., Gnanakaran, S., and Dale, B. E. (2011) Restructuring the crystalline cellulose hydrogen bond network enhances its depolymerization rate. *J Am Chem Soc* **133**, 11163-11174
6. Igarashi, K., Uchihashi, T., Koivula, A., Wada, M., Kimura, S., Okamoto, T., Penttila, M., Ando, T., and Samejima, M. (2011) Traffic jams reduce hydrolytic efficiency of cellulase on cellulose surface. *Science* **333**, 1279-1282
7. Cruys-Bagger, N., Tatsumi, H., Ren, G. R., Borch, K., and Westh, P. (2013) Transient kinetics and rate-limiting steps for the processive cellobiohydrolase Cel7A: effects of substrate structure and carbohydrate binding domain. *Biochemistry* **52**, 8938-8948
8. Cruys-Bagger, N., Elmerdahl, J., Praestgaard, E., Tatsumi, H., Spodsberg, N., Borch, K., and Westh, P. (2012) Pre-steady-state kinetics for hydrolysis of insoluble cellulose by cellobiohydrolase Cel7A. *The Journal of biological chemistry* **287**, 18451-18458
9. Knott, B. C., Haddad Momeni, M., Crowley, M. F., Mackenzie, L. F., Götz, A. W., Sandgren, M., Withers, S. G., Ståhlberg, J., and Beckham, G. T. (2014) The Mechanism of Cellulose Hydrolysis by a Two-Step, Retaining Cellobiohydrolase Elucidated by Structural and Transition Path Sampling Studies. *Journal of the American Chemical Society* **136**, 321-329
10. Fersht, A. (1985) *Enzyme structure and mechanism*, 2 ed., W.H. Freeman, San Francisco
11. Kurasin, M., and Valjamae, P. (2011) Processivity of cellobiohydrolases is limited by the substrate. *The Journal of biological chemistry* **286**, 169-177
12. Murphy, L., Cruys-Bagger, N., Damgaard, H. D., Baumann, M. J., Olsen, S. N., Borch, K., Lassen, S. F., Sweeney, M., Tatsumi, H., and Westh, P. (2012) Origin of initial burst in activity for Trichoderma reesei endo-glucanases hydrolyzing insoluble cellulose. *The Journal of biological chemistry* **287**, 1252-1260
13. Christensen, S. J., Kari, J., Badino, S. F., Borch, K., and Westh, P. (2018) Rate-limiting step and substrate accessibility of cellobiohydrolase Cel6A from Trichoderma reesei. *The FEBS journal* **285**, 4482-4493
14. Cruys-Bagger, N., Elmerdahl, J., Praestgaard, E., Tatsumi, H., Spodsberg, N., Borch, K., and Westh, P. (2012) Pre-steady state kinetics for the hydrolysis of insoluble cellulose by cellobiohydrolase Cel7A. *The Journal of biological chemistry* **287**
15. Igarashi, K., Koivula, A., Wada, M., Kimura, S., Penttila, M., and Samejima, M. (2009) High speed atomic force microscopy visualizes processive movement of Trichoderma reesei cellobiohydrolase I on crystalline cellulose. *The Journal of biological chemistry* **284**, 36186-36190
16. Kari, J., Kont, R., Borch, K., Buskov, S., Olsen, J. P., Cruyz-Bagger, N., Valjamae, P., and Westh, P. (2017) Anomeric Selectivity and Product Profile of a Processive Cellulase. *Biochemistry* **56**, 167-178

17. Fromm, S. J., and Fromm, H. J. (1999) A Two-Step Computer-Assisted Method for Deriving Steady-State Rate Equations. *Biochemical and Biophysical Research Communications* **265**, 448-452
18. Praestgaard, E., Elmerdahl, J., Murphy, L., Nymand, S., McFarland, K. C., Borch, K., and Westh, P. (2011) A kinetic model for the burst phase of processive cellulases. *The FEBS journal* **278**, 1547-1560
19. Kari, J., Olsen, J., Borch, K., Cruys-Bagger, N., Jensen, K., and Westh, P. (2014) Kinetics of Cellobiohydrolase (Cel7A) Variants with Lowered Substrate Affinity. *The Journal of biological chemistry* **289**, 32459-32468
20. Silveira, M. H. L., Aguiar, R. S., Siika-aho, M., and Ramos, L. P. (2014) Assessment of the enzymatic hydrolysis profile of cellulosic substrates based on reducing sugar release. *Bioresource Technology* **151**, 392-396
21. Sorensen, T. H., Cruys-Bagger, N., Windahl, M. S., Badino, S. F., Borch, K., and Westh, P. (2015) Temperature Effects on Kinetic Parameters and Substrate Affinity of Cel7A Cellobiohydrolases. *The Journal of biological chemistry* **290**, 22193-22202
22. Christensen, S. J., Krogh, K. B. R. M., Spodsberg, N., Borch, K., and Westh, P. (2019) A biochemical comparison of fungal GH6 cellobiohydrolases. *Biochem J* **476**, 2157-2172
23. Kari, J., Olsen, J. P., Jensen, K., Badino, S. F., Krogh, K. B. R. M., Borch, K., and Westh, P. (2018) Sabatier Principle for Interfacial (Heterogeneous) Enzyme Catalysis. *ACS Catalysis* **8**, 11966-11972
24. Schiano-di-Cola, C., Rojel, N., Jensen, K., Kari, J., Sorensen, T. H., Borch, K., and Westh, P. (2019) Systematic deletions in the cellobiohydrolase (CBH) Cel7A from the fungus Trichoderma reesei reveal flexible loops critical for CBH activity. *The Journal of biological chemistry* **294**, 1807-1815
25. Badino, S. F., Christensen, S. J., Kari, J., Windahl, M. S., Hvidt, S., Borch, K., and Westh, P. (2017) Exo-exo synergy between Cel6A and Cel7A from Hypocrea jecorina: Role of carbohydrate binding module and the endo-lytic character of the enzymes. **114**, 1639-1647
26. Christensen, S. J., Badino, S. F., Cavaleiro, A. M., Borch, K., and Westh, P. (2020) Functional analysis of chimeric TrCel6A enzymes with different carbohydrate binding modules. *Protein Engineering, Design and Selection*
27. Kari, J., Andersen, M., Borch, K., and Westh, P. (2017) An Inverse Michaelis–Menten Approach for Interfacial Enzyme Kinetics. *ACS Catalysis* **7**, 4904-4914
