# Supplementary material for: Physical constraints and functional plasticity of cellulases
Source: Nat Commun. 2021 Jun 22;12:3847. doi: 10.1038/s41467-021-24075-y (PMC8219668; doi:10.1038/s41467-021-24075-y)
Supplement: Supplementary file 3 — Reporting summary [file 41467_2021_24075_MOESM3_ESM.pdf]

nature research

Corresponding author(s): Peter Westh
Last updated by author(s): Apr 15, 2021

Reporting Summary

Nature Research wishes to improve the reproducibility of the work that we publish. This form provides structure for consistency and transparency in reporting. For further information on Nature Research policies, see our Editorial Policies and the Editorial Policy Checklist.

Statistics

For all statistical analyses, confirm that the following items are present in the figure legend, table legend, main text, or Methods section.

n/a	Confirmed	
☐	☒	The exact sample size (*n*) for each experimental group/condition, given as a discrete number and unit of measurement
☒	☐	A statement on whether measurements were taken from distinct samples or whether the same sample was measured repeatedly
☒	☐	The statistical test(s) used AND whether they are one- or two-sided<br>*Only common tests should be described solely by name; describe more complex techniques in the Methods section.*
☒	☐	A description of all covariates tested
☐	☒	A description of any assumptions or corrections, such as tests of normality and adjustment for multiple comparisons
☐	☒	A full description of the statistical parameters including central tendency (e.g. means) or other basic estimates (e.g. regression coefficient) AND variation (e.g. standard deviation) or associated estimates of uncertainty (e.g. confidence intervals)
☒	☐	For null hypothesis testing, the test statistic (e.g. *F*, *t*, *r*) with confidence intervals, effect sizes, degrees of freedom and *P* value noted<br>*Give P values as exact values whenever suitable.*
☒	☐	For Bayesian analysis, information on the choice of priors and Markov chain Monte Carlo settings
☒	☐	For hierarchical and complex designs, identification of the appropriate level for tests and full reporting of outcomes
☐	☒	Estimates of effect sizes (e.g. Cohen's *d*, Pearson's *r*), indicating how they were calculated

Our web collection on statistics for biologists contains articles on many of the points above.

Software and code

Policy information about availability of computer code

Data collection	Kinetic data was obtained from absorbance measurements which was done on a microplate reader (SpectraMax M2e, Molecular Devices, Sunnyvale, CA)
Data analysis	Analysis of enzyme kinetics was done using the software Origin pro v. 7 (OriginLab, Northampton, MA)<br>MD simulations was done using the following open-source software: GROMACS 2018.6, Modeller 9.23, Autodock Vina 1.1.2

For manuscripts utilizing custom algorithms or software that are central to the research but not yet described in published literature, software must be made available to editors and reviewers. We strongly encourage code deposition in a community repository (e.g. GitHub). See the Nature Research guidelines for submitting code & software for further information.

Data

Policy information about availability of data

All manuscripts must include a data availability statement. This statement should provide the following information, where applicable:
- Accession codes, unique identifiers, or web links for publicly available datasets
- A list of figures that have associated raw data
- A description of any restrictions on data availability

All data generated or analysed during this study are included in this published article (and its supplementary information files).

Field-specific reporting

Please select the one below that is the best fit for your research. If you are not sure, read the appropriate sections before making your selection.

☒ Life sciences ☐ Behavioural & social sciences ☐ Ecological, evolutionary & environmental sciences

For a reference copy of the document with all sections, see nature.com/documents/nr-reporting-summary-flat.pdf

Life sciences study design

All studies must disclose on these points even when the disclosure is negative.

Sample size	We characterized 83 enzymes (cellulases). The decision to use this sample size was based on two main factors: 1 - Our objective was to characterize as many representatives of each fungal cellulase type as possible; 2 - The number of enzymes was limited by availability, as all enzymes had to be expressed and purified in the same system. To our knowledge, this sample size is greater than in any other systematic study of this class of enzymes.
Data exclusions	Out of the 83 characterized enzymes, 6 of them were excluded from the regression shown in Fig. 2. The 95% prediction band was employed to guide our decision on which enzymes should be excluded. Given our objective of finding physicals limits for the action of these enzymes, this decision was rational, as less efficient enzymes will always exist, and including them in the regressions would contradict the very logical argument of this manuscript.
Replication	Almost all enzymes were subjected to a prior cycle of characterization. The enzymes that were previously characterized by our group or by other scientific reports had their kinetic parameters compared to the ones obtained in this study.
Randomization	The order of the characterizations of the enzymes did not follow a logical order.
Blinding	*Describe whether the investigators were blinded to group allocation during data collection and/or analysis. If blinding was not possible, describe why OR explain why blinding was not relevant to your study.*

Reporting for specific materials, systems and methods

We require information from authors about some types of materials, experimental systems and methods used in many studies. Here, indicate whether each material, system or method listed is relevant to your study. If you are not sure if a list item applies to your research, read the appropriate section before selecting a response.

Materials & experimental systems

n/a	Involved in the study	
☒	☐	Antibodies
☒	☐	Eukaryotic cell lines
☒	☐	Palaeontology and archaeology
☒	☐	Animals and other organisms
☒	☐	Human research participants
☒	☐	Clinical data
☒	☐	Dual use research of concern

Methods

n/a	Involved in the study	
☒	☐	ChIP-seq
☒	☐	Flow cytometry
☒	☐	MRI-based neuroimaging
